# Isolation of Antigen-Specific Nanobodies From Synthetic Libraries Using a Protein Selection Strategy That Combines MACS-Based Screening of YSD and FLI-TRAP

**DOI:** 10.21769/BioProtoc.5570

**Published:** 2026-01-20

**Authors:** Apisitt Thaiprayoon, Yodpong Chantarasorn, Worrapoj Oonanant, Anongnard Kasorn, Phoomintara Longsompurana, Satita Tapaneeyakorn, Pinpunya Riangrungroj, Fabien Loison, Andrew C. Kruse, Matthew P. DeLisa, Dujduan Waraho-Zhmayev

**Affiliations:** 1 Biological Engineering Program, Faculty of Engineering, King Mongkut's University of Technology Thonburi, Bangkok, Thailand; 2 Division of Ophthalmology, Faculty of Medicine, Vajira Hospital, Navamindradhiraj University, Bangkok, Thailand; 3 Department of Basic Medical Science, Faculty of Medicine Vajira Hospital, Navamindradhiraj University, Bangkok, Thailand; 4 National Nanotechnology Center (NANOTEC), National Science and Technology Development Agency (NSTDA), Thailand Science Park, Pathumthani, Thailand; 5 National Center for Genetic Engineering and Biotechnology (BIOTEC), National Science and Technology Development Agency (NSTDA), Pathumthani, Thailand; 6 Department of Microbiology, Faculty of Science, Mahidol University, Bangkok, Thailand; 7 Department of Biological Chemistry and Molecular Pharmacology, Harvard Medical School, Boston, MA, USA; 8 Robert F. Smith School of Chemical and Biomolecular Engineering, Cornell University, Ithaca, NY, USA; 9 Cornell Institute of Biotechnology, Cornell University, Ithaca, NY, USA

**Keywords:** FLI-TRAP, MACS-based yeast display, Nanobody screening, Protein–protein interaction, Twin-arginine translocation pathway

## Abstract

Although protein–protein interactions (PPIs) are central to nearly all biological processes, identifying and engineering high-affinity intracellular binders remains a significant challenge due to the complexity of the cellular environment and the folding constraints of proteins. Here, we present a two-stage complementary platform that combines magnetic-activated cell sorting (MACS)-based yeast surface display with functional ligand-binding identification by twin-arginine translocation (Tat)-based recognition of associating proteins (FLI-TRAP), a bacterial genetic selection system for efficient screening, validation, and optimization of PPIs. In the first stage, MACS-based yeast display enables the rapid high-throughput identification of candidate binders for a target antigen from a large synthetic-yeast display library through extracellular interaction screening. In the second stage, an antigen-focused library is subcloned into the FLI-TRAP system, which exploits the hitchhiker export process of the *Escherichia coli* Tat pathway to evaluate binder–antigen binding in the cytoplasm. This stage is achieved by co-expressing a Tat signal peptide–tagged protein of interest with a β-lactamase-tagged antigen target, such that only binder–antigen pairs with sufficient affinity are co-translocated into the periplasm, thus rendering the bacterium β-lactam antibiotic resistant. Because Tat-dependent export requires fully folded and soluble proteins, FLI-TRAP further serves as a stringent in vivo filter for intracellular compatibility, folding, and stability. Therefore, this approach provides a powerful and cost-effective pipeline for discovering and engineering intracellular protein binders with high affinity, specificity, and functional expression in bacterial systems. This workflow holds promise for several applications, including synthetic biology and screening of theragnostic proteins and PPI inhibitors.

Key features

• Combines a single round of MACS enrichment with FLI-TRAP for high-throughput Nb discovery.

• Reduces time and resource demands compared to traditional workflows involving multiple rounds of MACS/FACS.

• Enables in vivo selection of high-affinity, functional binders via Tat-dependent export linked to β-lactam resistance, correlating binding affinity and solubility with antibiotic resistance.

## Background

The biologics market is one of the fastest-growing sectors in the pharmaceutical industry, with over 100 antibody-based therapeutics now approved by the U.S. Food and Drug Administration (FDA) and/or the European Medicines Agency [1]. While full-length monoclonal antibodies (mAbs) remain the dominant form, other antibody derivatives have also gained regulatory approval, including fragment antigen-binding regions, single-chain variable fragments, and single-domain antibodies [also called variable domain of the heavy chain antibodies or nanobodies (Nbs)] [2]. Among these, Nbs derived from camelid species, such as camels, llamas, and alpacas, offer unique advantages due to their small size, high solubility, and exceptional stability. Since the FDA approved the first therapeutic Nb (caplacizumab) in 2019, Nbs have become increasingly valuable in both preclinical research and therapeutic development [3]. Their extended complementarity-determining region 3 enables them to access hidden or concave epitopes that are often inaccessible to conventional mAbs, while their small size facilitates microbial expression and simplifies downstream engineering [4].

**Figure 1.**
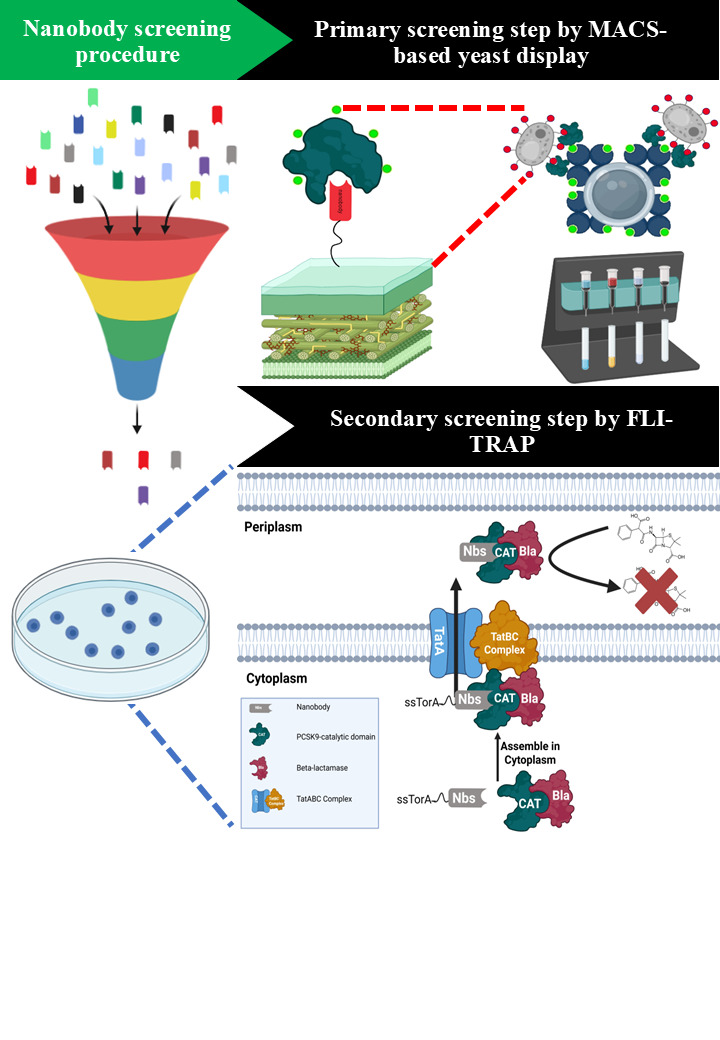
Schematic representation of the integrated platform for the isolation of PCSK9-specific nanobodies (Nbs) via combination of MACS-based yeast display and FLI-TRAP technique. A synthetic nanobody library is displayed on the surface of *Saccharomyces cerevisiae* BJ5465 and subjected to antigen-specific enrichment using MACS with recombinant PCSK9. This step selectively enriches yeast clones expressing Nbs with binding activity toward PCSK9. The enriched Nb population is subsequently transferred to a bacterial functional screening system based on functional ligand-binding identification by twin-arginine translocation (Tat)-based recognition of associating proteins (FLI-TRAP). In the FLI-TRAP system, Nb candidates are expressed in the cytoplasm of *Escherichia coli* as N-terminal fusions to the Tat signal peptide derived from trimethylamine N-oxide reductase (ssTorA), whereas PCSK9 is expressed as a fusion with the reporter enzyme β-lactamase (Bla). Productive Nb–PCSK9 interactions in the cytoplasm result in the formation of a stable protein complex that is specifically recognized by the Tat translocase. The Tat pathway mediates the translocation of fully folded proteins and protein complexes across the inner membrane into the periplasm; consequently, only correctly folded and sufficiently stable Nb–PCSK9–Bla complexes are exported. Periplasmic localization of Bla confers resistance to β-lactam antibiotics, thereby enabling direct in vivo selection of PCSK9-specific Nbs with favorable binding affinity and solubility based on bacterial survival under antibiotic selection pressure.

**Figure 2.**
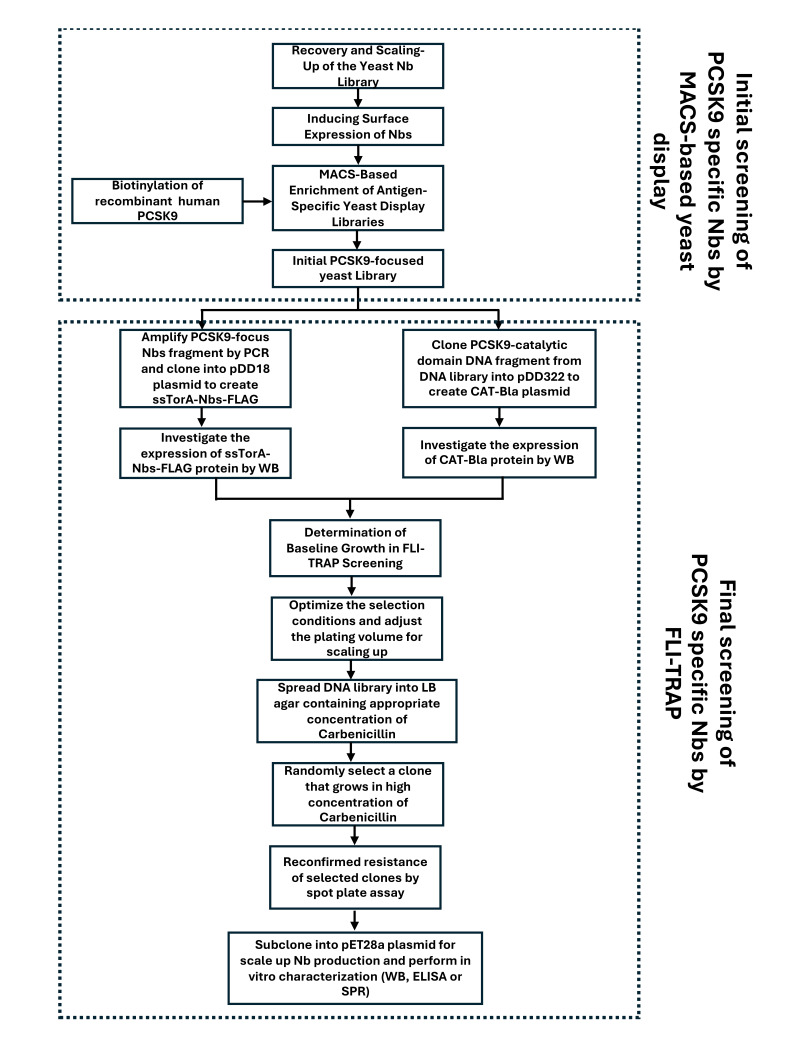
Critical steps in the combined magnetic-activated cell sorting (MACS)-based yeast display and functional ligand-binding identification by twin-arginine translocation (Tat)-based recognition of associating proteins (FLI-TRAP) workflow for screening PCSK9-specific nanobodies (Nbs).

Efficient and scalable platforms for Nb discovery are crucial for exploiting these properties. While being effective, conventional selection strategies, such as multiple rounds of magnetic-activated cell sorting (MACS) and fluorescence-activated cell sorting (FACS), are time-consuming and resource-intensive. In contrast, we recently demonstrated that a hybrid approach combining MACS-based yeast display with bacterial functional ligand-binding identification by twin-arginine translocation (Tat)-based recognition of associating proteins (FLI-TRAP) can significantly streamline the Nb discovery process [5,6]. We successfully isolated three highly specific Nbs against the catalytic domain of human proprotein convertase subtilisin/kexin type 9 (PCSK9) using only a single round of MACS pre-enrichment followed by a single round of FLI-TRAP selection [7]. This approach represents a significant improvement over traditional methods, which typically require 5–6 rounds of MACS and 1–2 rounds of FACS to achieve similar specificity. These findings highlight the high-throughput and cost-effective nature of combining MACS-based yeast display with FLI-TRAP (Figures 1 and 2).

FLI-TRAP leverages the unique capability of the Tat pathway in *Escherichia coli*, which exports fully folded and multimeric protein complexes from the cytoplasm to the periplasm. By fusing the Tat signal peptide of *E. coli* trimethylamine *N*-oxide reductase (ssTorA) to the Nb candidate (X) and a reporter protein [β-lactamase (Bla)] to the target antigen (Y), the successful protein–protein interaction (PPI) between X and Y enables the export of the complex into the periplasm, conferring resistance to β-lactam antibiotics such as ampicillin and carbenicillin. Notably, the degree of resistance correlates with binding affinity and/or solubility, allowing direct in vivo selection of high-affinity binders from focused libraries [5]. However, FLI-TRAP is limited by its reliance on soluble target proteins in the cytoplasm; insoluble proteins cannot be efficiently exported via the Tat pathway. This limitation can be addressed by expressing only the soluble fragments or functional domains of the target protein during screening [8,9].

Together, the combination of MACS-based yeast display for broad antigen-binding enrichment and FLI-TRAP for functional bacterial selection offers a powerful, rapid, and economical platform for Nb discovery. This two-step strategy reduces the need for iterative screening and enables efficient identification of binders with strong biological relevance, making it an attractive alternative to traditional methods.

## Materials and reagents

**Bacterial strains**

1. Competent BL21(DE3) *E. coli* cells (New England Biolabs, catalog number: C2527H)

2. Competent NEB10β *E. coli* cells (New England Biolabs, catalog number: C3019H)

3. *Saccharomyces cerevisiae* strain BJ5465 (American Type Culture Collection, catalog number: 208289)

**Reagents**

1. Sodium citrate (Merck Millipore, CAS: 6132-04-03)

2. Yeast nitrogen base w/o amino acids (USBio, CAS: 38210000)

3. Glucose (Merck Millipore, CAS: 50-99-77)

4. Drop out mix synthetic minus tryptophan w/o yeast nitrogen base (USBio, catalog number: D9530)

5. Penicillin/streptomycin solution (Gibco, catalog number: 1TFS-1CC-15140122)

6. Galactose (Merck Millipore, CAS: 59-23-4)

7. Peptone (Himedia, catalog number: RM001-500G)

8. Yeast extract (Himedia, catalog number: RM027-500G)

9. Sodium chloride (Merck Millipore, CAS: 7647-14-5)

10. Agar (Himedia, CAS: GRM026)

11. Chloramphenicol (Merck Millipore, CAS: 56-75-7)

12. Kanamycin (Merck Millipore, CAS: 25389-94-0)

13. Carbenicillin (Sigma-Aldrich, CAS: 4800-94-6)

14. Arabinose (Merck Millipore, CAS: 5238-37-0)

15. Bacto-tryptone (Himedia, catalog number: M014-500G)

16. Dimethyl sulfoxide (DMSO) (Merck Millipore, CAS: 67-8-5)

17. Potassium chloride (Merck Millipore, CAS: 7447-40-7)

18. Magnesium sulfate (Merck Millipore, CAS: 7487-88-9)

19. Tween-20 (Merck Millipore, CAS: 9005-64-5)

20. Tris base (Merck Millipore, CAS: 77-68-1)

21. Sodium dodecyl sulfate (SDS) (Merck Millipore, CAS: 151-21-3)

22. 40% Acrylamide/Bis solution (Bio-Rad, CAS: 1610140)

23. Ammonium persulfate (APS) (Merck Millipore, CAS: 7727-54-0)

24. Bromophenol blue (Merck Millipore, CAS: 115-39-9)

25. Glycerol (Merck Millipore, CAS: 56-81-5)

26. β-mercaptoethanol (Merck Millipore, CAS: 60-24-2)

27. Ethylenediaminetetraacetic acid (EDTA) (Merck Millipore, CAS: 60-00-04)

28. Tetramethylethylenediamine (TEMED) (Merck Millipore, CAS: 110-18-9)

29. EZ-Link^TM^ Sulfo-NHS-Biotin (Thermo Scientific^TM^, catalog number: 21217)

30. Sodium phosphate dibasic (Merck Millipore, CAS: 7558-79-4)

31. Potassium phosphate monobasic (Merck Millipore, CAS: 7778-77-0)

32. Acetic acid (Merck Millipore, CAS: 64-19-7)

33. HA Tag recombinant mouse monoclonal antibody (2-2.2.14), Alexa Fluor^TM^ Plus 488 (Thermo Fischer Scientific, catalog number: 740005MP48820UG)

34. HA Tag recombinant mouse monoclonal antibody (2-2.2.14), Alexa Fluor^TM^ Plus 647 (Thermo Fischer Scientific, catalog number: 740005MP64720UG)

35. Zymolyase (Zymo Research, catalog number: E1004)

36. Quick Start^TM^ Bradford protein assay (Bio-Rad, catalog number: 5000201)

37. Hydrochloric acid (HCl) (Merck Millipore, CAS: 258148)

38. Sodium hydroxide (NaOH) (Merck Millipore, CAS: 221465)

39. HRP Anti-HA tag antibody (Abcam, catalog number: 173826)

40. EasySep^TM^ Direct Human PBMC Isolation kit (STEMCELL Technologies, catalog number: 19654)

41. Clarity Max Western ECL substrate (Bio-Rad, catalog number: 1705062)

42. Recombinant Human PCSK9 protein (His & AVI Tag), biotinylated, HPLC-verified (Sino Biological, catalog number: 29698-H27H-B)

43. xbaI restriction enzyme (Bio-Rad, catalog number: R0145S)

44. SalI-HF restriction enzyme (Bio-Rad, catalog number: R3138S)

45. AvrII restriction enzyme (Bio-Rad, catalog number: R0174S)

46. NoTI-HF restriction enzyme (Bio-Rad, catalog number: R3189S)

47. NucleoSpin Plasmid Mini kit for plasmid DNA (MACHEREY-NAGEL, catalog number: 740588.50)

48. NucleoSpin Gel and PCR Clean-up Mini kit for gel extraction or PCR clean up (MACHEREY-NAGEL, catalog number: 740609.50)

49. T4 DNA ligase (Bio-Rad, catalog number: M0202S)

50. Anti-FLAG-HRP (HRP Anti-DDDDK tag, binds to FLAG^®^ tag sequence) antibody (M2) (Abcam, catalog number: ab49763)

51. Anti-Beta lactamase antibody (Abcam, catalog number: ab12251)

52. Goat anti-mouse IgG secondary antibody (HRP) (Sino Biological, catalog number: SSA007)

53. Absolute ethanol (Merck Millipore, CAS: 64-17-5)

54. Blotting-grade blocker (Bio-Rad, catalog number: 1706404)

**Solutions**

1. Yglc4.5–Trp medium (see Recipes)

2. Yglc–Gal pH 6.0 (see Recipes)

3. Luria-Bertani (LB) broth (see Recipes)

4. LB agar (see Recipes)

5. Chloramphenicol solution (see Recipes)

6. Kanamycin solution (see Recipes)

7. Carbenicillin solution (see Recipes)

8. Arabinose solution (see Recipes)

9. Super optimal broth (SOB) medium (see Recipes)

10. Phosphate buffer saline (PBS) (see Recipes)

11. Western blot blocking buffer (see Recipes)

12. Phosphate buffer saline with Tween-20 (PBST) (see Recipes)

13. 0.5 M Tris pH 6.8 solution (see Recipes)

14. 1.5 M Tris pH 8.8 solution (see Recipes)

15. 10% SDS solution (see Recipes)

16. 10% ammonium persulfate (APS) (see Recipes)

17. 6× Laemmli sample loading buffer (see Recipes)

18. 12% SDS-polyacryalmide gel (see Recipes)

19. 6% SDS-polyacrylamide gel (see Recipes)

20. 50× Tris-acetate-EDTA buffer (TAE) (see Recipes)

21. Cell lysis buffer (see Recipes)

22. 10% APS (see Recipes)

23. Yeast extract peptone dextrose agar (see Recipes)

**Recipes**

**1. Yglc4.5–Trp medium**

**Table:** 

Reagent	Final concentration	Quantity or volume
Sodium citrate	80 mM	20.6448 g
Yeast nitrogen base w/o amino acids	6.7 g/L	6.7 g
Glucose	2% w/v	20 g
Drop out mix synthetic minus tryptophan w/o yeast nitrogen base	3.8 g/L	3.8 g
Penicillin/streptomycin solution	1×	10 mL
H_2_O	n/a	To 1,000 mL

Adjust pH to 4.5 with 1 N HCl and sterilize by autoclaving.

**2. Yglc–Gal pH 6.0**

**Table:** 

Reagent	Final concentration	Quantity or volume
Yeast nitrogen base w/o amino acids	6.7 g/L	6.7 g
Galactose	2% w/v	20 g
Drop out mix synthetic minus tryptophan w/o yeast nitrogen base	3.8 g/L	3.8 g
Penicillin/streptomycin solution	1×	10 mL
H_2_O	n/a	To 1,000 mL

Adjust pH to 4.5 with 1 N HCl and sterilize by autoclaving.

**3. LB broth**

**Table:** 

Reagent	Final concentration	Quantity or volume
Peptone	10 g/L	10 g
Yeast extract	10 g/L	10 g
Sodium chloride	5 g/L	5 g
H_2_O	n/a	To 1,000 mL

Adjust pH to 7.4 with 1 N HCl and sterilize by autoclaving.

**4. LB agar**

**Table:** 

Reagent	Final concentration	Quantity or volume
Peptone	10 g/L	10 g
Yeast extract	10 g/L	10 g
Sodium chloride	5 g/L	5 g
Agar	15 g/L	15 g
H_2_O	n/a	To 1,000 mL

Adjust pH to 7.4 with 1 N HCl and sterilize by autoclaving.

**5. Chloramphenicol solution**

**Table:** 

Reagent	Final concentration	Quantity or volume
Chloramphenicol	34 g/L	340 mg
Absolute ethanol	n/a	To 10 mL

Sterilize by filtering with a 0.22 μm filter. Aliquot in a 1 mL sterile microtube and store at -20 °C.

**6. Kanamycin solution**

**Table:** 

Reagent	Final concentration	Quantity or volume
Kanamycin	50 g/L	500 mg
H_2_O	n/a	To 10 mL

Sterilize by filtering with a 0.22 μm filter. Aliquot in a 1 mL microtube and store at -20 °C.

**7. Carbenicillin solution**

**Table:** 

Reagent	Final concentration	Quantity or volume
Carbenicillin	100 g/L	1 g
H_2_O	n/a	To 10 mL

Sterilize by filtering through a 0.22 μm filter. Aliquot 1 mL into sterile amber microtubes and store at -20 °C.

**8. Arabinose solution**

**Table:** 

Reagent	Final concentration	Quantity or volume
Arabinose	20% w/v	20 g
H_2_O	n/a	To 100 mL

Sterilize by filtering through a 0.22 μm filter. Aliquot 1 mL into sterile microtubes and store at -20 °C.

**9. SOB medium**

**Table:** 

Reagent	Final concentration	Quantity or volume
Bacto-tryptone	20 mg/mL	20 g
Yeast extract	10 mg/mL	10 g
Sodium chloride	5 mg/mL	5 g
Potassium chloride	2.5 mM	0.186 g
Magnesium sulfate	20 mM	2.4 g
H_2_O	n/a	To 1,000 mL

Adjust the pH to 7.4 with 1 N HCl and sterilize by autoclaving.

**10. PBS**

**Table:** 

Reagent	Final concentration	Quantity or volume
Sodium chloride	0.137 M	8 g
Potassium chloride	2.7 mM	0.2 g
Sodium phosphate dibasic	10 mM	1.44 g
Potassium phosphate monobasic	1.8 mM	0.245 g
H_2_O	n/a	To 1,000 mL

Adjust pH to 7.4 with 1 N HCl and sterilize by autoclaving.

**11. Western blot blocking buffer**

**Table:** 

Reagent	Final concentration	Quantity or volume
Sodium chloride	0.137 M	8 g
Potassium chloride	2.7 mM	0.2 g
Sodium phosphate dibasic	10 mM	1.44 g
Potassium phosphate monobasic	1.8 mM	0.245 g
Blotting-grade blocker	5% w/v	50 g
H_2_O	n/a	To 1,000 mL

Adjust pH to 7.4 with 1 N HCl and sterilize by autoclaving.

**12. PBST**

**Table:** 

Reagent	Final concentration	Quantity or volume
Sodium chloride	0.137 M	8 g
Potassium chloride	2.7 mM	0.2 g
Sodium phosphate dibasic	10 mM	1.44 g
Potassium phosphate monobasic	1.8 mM	0.245 g
Tween-20	0.1% v/v	1 mL
H_2_O	n/a	To 1,000 mL

Adjust pH to 7.4 with 1 N HCl and sterilize by autoclaving.

**13. 0.5 M Tris pH 6.8**

**Table:** 

Reagent	Final concentration	Quantity or volume
Tris base	0.5 M	18.2 g
H_2_O	n/a	To 100 mL

Adjust pH to 6.8 with 1 N HCl and sterilize by autoclaving.

**14. 1.5 M Tris pH 8.8**

**Table:** 

Reagent	Final concentration	Quantity or volume
Tris base	0.5 M	12.1 g
H_2_O	n/a	To 100 mL

Adjust pH to 8.8 with 1 N HCl and sterilize by autoclaving.

**15. 10% SDS solution**

**Table:** 

Reagent	Final concentration	Quantity or volume
SDS	10% w/v	10 g
H_2_O	n/a	To 100 mL

**16. 10% APS**

**Table:** 

Reagent	Final concentration	Quantity or volume
APS	10% w/v	10 g
H_2_O	n/a	To 100 mL

**17. 6× Laemmli sample loading buffer**

**Table:** 

Reagent	Final concentration	Quantity or volume
SDS	10% w/v	1 g
Bromophenol blue	0.01% v/v	10 mg
Glycerol	50% w/v	5 mL
Tris base	0.5 M	0.6 g
β-mercaptoethanol	10% v/v	1 mL
H_2_O	n/a	To 10 mL

**18. 12% SDS polyacrylamide gel**

**Table:** 

Reagent	Quantity or volume
40% acrylamide/bis solution	2.4 mL
1.5 M Tris pH 8.8	2 mL
10% SDS	80 μL
10% APS	80 μL
TEMED	8 μL (0.1% v/v)
H_2_O	To 8 mL

**19. 6% SDS polyacrylamide gel**

**Table:** 

Reagent	Quantity or volume
40% acrylamide/bis solution	0.75 mL
1.5 M Tris pH 8.8	1.25 mL
10% SDS	50 μL
10% APS	50 μL
TEMED	5 μL (0.1% v/v)
H_2_O	To 5 mL

**20. 50× TAE**

**Table:** 

Reagent	Final concentration	Quantity or volume
Tris base	2 M	242.2 g
Acetic acid	1 M	57.1 mL
EDTA	50 mM	18.612 g
H_2_O	n/a	To 1,000 mL

**21. Cell lysis buffer**

**Table:** 

Reagent	Final concentration	Quantity or volume
Sodium chloride	0.137 M	8 g
Potassium chloride	2.7 mM	0.2 g
Sodium phosphate dibasic	10 mM	1.44 g
Potassium phosphate monobasic	1.8 mM	0.245 g
SDS	1% w/v	10 g
H_2_O	n/a	To 1,000 mL

Adjust pH to 7.4 with 1 N HCl and sterilize by autoclaving.

**22. 10% APS**

**Table:** 

Reagent	Final concentration	Quantity or volume
APS	10% w/v	10 ml
H_2_O	n/a	To 100 mL

**23. Yeast extract peptone dextrose agar (YPD)**

**Table:** 

Reagent	Final concentration	Quantity or volume
Yeast extract	10 g/L	10 g
Peptone	20 g/L	20 g
Glucose	20 g/L	20 g
Agar	15 g/L	15 g
H_2_O	n/a	To 1000 mL

**Laboratory supplies**

1. DURAN^®^ culture flask, Erlenmeyer shape, straight neck, 2,000 mL (DWK Life Sciences, catalog number: 217716309)

2. DURAN^®^ culture flask, Erlenmeyer shape, straight neck, 200 mL (DWK Life Sciences, catalog number: 217713209)

3. Falcon^®^ 100 mm × 15 mm not TC-treated bacteriological Petri dish (Corning, catalog number: 351029)

4. Axygen^®^ 1.5 mL snap-lock microcentrifuge tube, polypropylene, clear, sterile (Corning, catalog number: MCT-150-C-S)

5. Corning^®^ polycarbonate 1–2 mL cryogenic vial storage box, holds 81 vials (Corning, catalog number: 431119)

6. Corning^®^ 2 mL external threaded polypropylene cryogenic vial with 1D and 2D bar codes (Corning, catalog number: 8671)

7. Corning^®^ DeckWorks 0.1–10 µL low-binding pipette tips, graduated, hinged racks, natural, nonsterile, polypropylene (Corning, catalog number: 4147)

8. Corning^®^ DeckWorks 1–200 µL low-binding pipette tips, graduated, hinged racks, natural, nonsterile, polypropylene (Corning, catalog number: 4148)

9. Corning^®^ DeckWorks 1–300 µL low-binding pipette tips, graduated, hinged racks, natural, nonsterile, polypropylene (Corning, catalog number: 4149)

10. Polyvinylidene difluoride (PVDF) western blotting membrane (Merck Millipore, CAS: 03010040001)

11. 0.22 µm filter membranes, nitrocellulose (Merck Millipore, CAS: Z358657)

12. Gene pulser/MicroPulser electroporation cuvettes, 0.1 cm gap (Bio-Rad, catalog number: 1652083)

## Equipment

1. Incubator shaker (RADOBIO, catalog number: MS350T)

2. Multiskan SkyHigh microplate spectrophotometer (Thermo Fischer Scientific, catalog number: A51119500C)

3. Sorvall^TM^ Legend^TM^ Micro 21R microcentrifuge (Thermo Fischer Scientific, catalog number: 75002544)

4. Pipet-Lite LTS Pipette L-2XLS+ manual single-channel pipette, 0.1–2 μL (Mettler Toledo, catalog number: 17014393)

5. Pipet-Lite LTS Pipette L-20XLS+ manual single-channel pipette, 2–20 μL (Mettler Toledo, catalog number: 17014392)

6. Pipet-Lite LTS Pipette L-20XLS+ manual single-channel pipette, 10–100 μL (Mettler Toledo, catalog number: 17014384)

7. Pipet-Lite LTS Pipette L-20XLS+ manual single-channel pipette, 100–1,000 μL (Mettler Toledo, catalog number: 17014382)

8. Pipet-Lite Multi Pipette L8-50XLS+ manual single-channel pipette, 5–50 μL (Mettler Toledo, catalog number: 17013804)

9. TSX Universal Series general purpose ultra-low freezers (Thermo Fischer Scientific, catalog number: TSX70086FA)

10. Attune Xenith flow cytometer (Thermo Fischer Scientific, catalog number: A59358)

11. Ultrasonic homogenizer (BUENO BIOTECH, model: BEM-150A)

12. Precision^TM^ general purpose baths (Thermo Fischer Scientific, catalog number: TSGP02)

13. EasySep^TM^ magnet (STEMCELL Technologies, catalog number: 18000)

14. ChemiDoc XRS+ system (Bio-Rad, catalog number: 1708265)

15. Heratherm^TM^ general protocol microbiological incubators (Thermo Fischer Scientific, catalog number: 51028063)

16. MicroPulser electroporator (Bio-Rad, catalog number: 1652100)

## Procedure

**A. Recovery and scaling up of the yeast Nb library**

*Note: This protocol was adapted from the one described by McMahon et al. [10].*

1. Thaw frozen aliquots of the yeast Nb library on ice, ensuring that the total number of recoverable yeast cells is at least five times greater than the diversity of the library. For the complete naïve library, this requires a minimum of 2.5 × 10^9^ viable cells.

2. Recover the yeast in a 2 L Erlenmeyer flask containing 1 L of Yglc4.5–Trp medium by shaking at 200 rpm at 30 °C overnight. Optionally, take a small aliquot of the library immediately after resuspension and plate it on yeast extract–peptone–dextrose (YPD) agar to assess library diversity.

3. Expand the culture to three 2 L Erlenmeyer flasks containing 1 L of Yglc4.5–Trp medium and continue incubating at 30 °C while shaking at 200 rpm for 48–72 h. Optical density at 600 nm (OD_600_) usually ranges between 10 and 30 at that point.

*Note: The OD_600_ of the yeast culture is used to estimate cell density. An OD_600_ of 1 corresponds to approximately 1.5 × 10^7^ yeast cells/mL.*

4. Centrifuge the cultures at 3,500× *g* for 5 min, then resuspend the cell pellets in Yglc4.5–Trp medium supplemented with 10% dimethyl sulfoxide (DMSO) to achieve a final concentration of 1 × 10^10^ cells per mL. Then, dispense 2 mL aliquots into cryovials and freeze at -80 °C using a controlled-rate cell freezing container. Each cryovial should contain a sufficient number of viable cells to regenerate the complete library upon thawing.

**B. Inducing surface expression of Nbs**

*Note: Nb expression is regulated by the galactokinase (*GAL1*) promoter, which is inducible and activated only in the presence of galactose. Consequently, Nbs are expressed on the surface of yeast cells only when they are cultured in a medium containing galactose as the primary carbon source. To initiate Nb display, yeast cells should first be grown in a raffinose-based medium (e.g., Yglc4.5–Trp), then transferred to a galactose-containing induction medium (Yglc–Gal, pH 6.0). This shift activates the* GAL1 *promoter, leading to production and surface display of Nbs for downstream applications such as screening, binding assays, or FACS.*

1. Thaw frozen aliquots of the yeast Nb library at 30 °C, ensuring that the total number of viable cells recovered is at least five times the library’s diversity. For the complete naïve library, a minimum of 2.5 × 10^9^ viable cells is required.

2. Inoculate the thawed yeast into a 2 L Erlenmeyer flask containing 1 L of Yglc4.5–Trp medium and incubate overnight at 30 °C with shaking at 200 rpm to allow recovery.

3. To induce Nb expression in the Nb Library, dilute a cell population at least tenfold greater than the library diversity into Yglc–Gal (pH 6.0) medium. Incubate the culture at 25 °C with shaking at 200 rpm for 48 h. Ensure the starting inoculum contains at least 5 × 10^9^ yeast cells to avoid losing any clones during passage.

**Critical:** Maintaining strict sterility throughout the selection process is vital. Unlike routine yeast culture, the cells will undergo multiple passages over several days, significantly increasing the risk of contamination. To minimize this risk, use sterile filter tips and freshly sterilized media for each selection step. Regularly disinfect hands, work surfaces, and pipettes with ethanol. While working within a sterile hood is ideal, a standard laboratory bench is acceptable provided that appropriate aseptic techniques and careful handling are consistently practiced.

**C. Assessing Nb expression by flow cytometry**

*Note: Before initiating any selection steps, it is recommended to verify that Nb expression has been successfully induced on the yeast cell surface. Confirming effective Nb expression ensures that subsequent binding assays and selections accurately reflect the functional display of Nbs. This validation step is typically performed using a rapid analytical flow cytometry assay, which quantitatively measures the presence of Nbs on the yeast cell surface. It involves staining the yeast cells with a fluorescently labeled anti-hemagglutinin (HA) antibody that recognizes an epitope tag fused to the Nb, allowing precise detection and quantification of Nb expression levels. By comparing stained samples to unstained controls, gating strategies can be established to distinguish expressing vs. non-expressing cell populations. This step provides a critical quality control checkpoint to confirm that the* GAL1 *promoter-driven induction was successful, thereby maximizing the reliability and interpretability of downstream selection experiments.*

1. Measure the cell density of the induced Nb library yeast culture, using the rule that an OD_600_ of 1 corresponds to approximately 1.5 × 10^7^ cells/mL.

2. Pipette approximately 1 × 10^6^ induced yeast cells into 500 μL of PBS in a microcentrifuge tube. Centrifuge the tubes at 3,500× *g* for 1 min at 4 °C to pellet the cells.

4. Carefully aspirate the supernatant without disturbing the pellet, then resuspend each cell pellet in 100 μL of fresh selection buffer.

5. In one tube, add approximately 0.5 μg of anti-HA antibody conjugated to Alexa Fluor 647 or Alexa Fluor 488 to detect Nb expression on the yeast surface. Additionally, add the protein of interest, labeled with a spectrally distinct fluorescent dye, at a final concentration of 1 μM to evaluate specific antigen binding vs. nonspecific interactions. Leave the second tube unstained to serve as a negative control.

6. Incubate both tubes on a rocking platform at 4 °C for 15 min to allow for antibody and antigen binding.

7. Centrifuge the tubes again at 3,500× *g* for 1 min at 4 °C to pellet the cells.

8. Aspirate the supernatant carefully, then resuspend each pellet in 500 μL of PBS to remove unbound antibody and antigen.

9. Repeat the centrifugation and aspiration step to thoroughly remove residual unbound reagents.

10. Finally, resuspend the cells in 100 μL of PBS and analyze Nb expression levels by flow cytometry. Use the unstained sample to set gating parameters, ensuring accurate distinction between positive and negative populations.

**D. Assessing Nb expression by western blotting**

*Note: If a flow cytometer or FACS instrument is unavailable, we recommend assessing Nb expression in the yeast library by western blotting as an alternative validation. This method involves lysing yeast cells and separating their total proteins by SDS-PAGE, followed by transferring the separated proteins to a membrane for immunodetection. Nb expression can be confirmed by probing the membrane with an antibody specific to the HA epitope tag fused to the Nb. Detection of an HA-tagged Nb band at the expected molecular weight provides qualitative confirmation of Nb expression. While western blotting does not offer single-cell resolution or quantitative data on surface display levels like flow cytometry, it remains a reliable and accessible technique to verify overall Nb expression within the yeast cell population. Implementing this approach as a complementary or alternative assay ensures that Nb expression has been successfully induced before proceeding with the selection or screening workflows.*

1. Determine the cell density of the induced Nb Library yeast cell culture, using the approximation that an OD_600_ of 1 corresponds to roughly 1.5 × 10^7^ cells/mL.

2. Add 500 μL of PBS to each of two microcentrifuge tubes.

3. Transfer approximately 1 × 10^7^ induced yeast cells into each tube. Then, centrifuge the tubes at 3,500× *g* for 1 min at 4 °C to pellet the cells.

4. Gently remove the supernatant without disturbing the pellet, then resuspend each pellet in 100 μL of fresh PBS.

5. Add 10 μg of lyophilized zymolyase to the yeast cell suspension and incubate in a 30 °C water bath for 1 h to enzymatically digest the yeast cell walls.

6. Lyse the yeast cells by sonication and centrifuge the lysate at 13,000× *g* for 10 min to separate the soluble protein fraction from the insoluble debris.

7. Discard the soluble fraction to remove the unwanted cytoplasmic fraction, as the Nb is expected to be present on the yeast cell surface. Collect the insoluble fraction and wash it three times with PBS to eliminate cytoplasmic contamination. Mix the washed insoluble fraction with cell lysis buffer (see Recipes) to disrupt the debris and release the Nbs integrated into the cell membrane.

8. Centrifuge the insoluble fraction again at 13,000× *g* for 15 min to ensure complete separation of the particulate material. Collect the resulting supernatant carefully and determine the protein concentration using the Bradford assay according to the manufacturer’s instructions.

9. Resuspend the remaining insoluble pellet in 6× Laemmli sample loading buffer (see Recipes). Heat the samples at 100 °C for 5 min in a water bath to denature the proteins.

10. Load 5 μg of the prepared insoluble protein sample onto a 10% SDS-PAGE gel and perform electrophoresis to separate the proteins by their molecular weight. Transfer the separated proteins onto a polyvinylidene difluoride membrane (Thermo Fisher Scientific) for western blot analysis. Probe the membrane with a horseradish peroxidase (HRP)-conjugated mouse anti-HA antibody (1:3,000 dilution) to detect HA-tagged Nbs. Visualize the signals using chemiluminescent ECL substrates and capture membrane images using a ChemiDoc XRS+ system to confirm Nb expression and assess protein integrity.

**E. MACS-based enrichment of antigen-specific yeast display libraries**

*Note: The EasySep^TM^ Human Blood Isolation kit can be effectively repurposed for MACS-based enrichment of antigen-specific clones from a yeast surface display library. By adapting the kit’s column, nonspecific or unbound yeast cells can be selectively depleted after incubation with a biotinylated antigen and streptavidin-conjugated magnetic particles. Yeast cells displaying Nbs that do not bind the antigen become magnetically labeled and eliminated during separation, while those expressing antigen-specific binders remain in the supernatant and are recovered for further enrichment or downstream applications. This approach enables rapid, high-purity, and viable enrichment of target-specific yeast clones without requiring specialized MACS columns, making it ideal for early-stage selection in Nb library screening workflows.*

1. Harvest 1 × 10^8^ induced yeast cells cultured under *GAL1*-inducing conditions to ensure surface Nb expression by centrifugation and resuspend them in 1 mL of sterile PBS. Transfer the suspension into a sterile EasySep^TM^ separation tube. Add 1–2 μg of the biotinylated target antigen to the yeast suspension and incubate at 37 °C with shaking at 200 rpm for 20 min to allow the specific binding of the Nbs to the target.

2. Add the EasySep^TM^ isolation cocktail directly to the tube and incubate the suspension again at 37 °C with shaking at 200 rpm for 20 min to optimize the binding conditions for the subsequent interaction with the RapidSpheres^TM^.

3. Vortex the streptavidin-coated RapidSpheres^TM^ magnetic beads vigorously for 1 min to ensure consistent suspension. Add 40 μL of RapidSpheres^TM^ per 1 mL of sample to the yeast–antigen mixture and incubate at 37 °C with shaking at 200 rpm for 20 min to allow for interactions between the beads and the biotinylated antigen bound to the yeast cells expressing specific Nbs.

4. As recommended by the manufacturer, top up the sample to 2 mL with EasySep^TM^ cell separation buffer and mix gently by pipetting 2–3 times to ensure homogeneity. Place the tube into the EasySep^TM^ magnet and incubate for 3 min at room temperature under static conditions. Remove the unbound yeast population (nonspecific binders or non-expressing clones) after magnetic immobilization.

5. Collect the enriched fraction containing antigen-specific yeast cells by carefully inverting the magnet and pouring the supernatant into a new sterile tube in a single continuous motion.

*Note: This adapted protocol provides a rapid, efficient, and column-free magnetic selection strategy for initial enrichment of antigen-specific yeast clones, facilitating downstream characterization and affinity maturation of candidate Nbs.*

**F. Protein biotinylation**

*Note: Biotin, a water-soluble B-complex vitamin (molecular weight: 244 Da), exhibits an exceptionally high affinity for the proteins avidin and streptavidin, forming one of the strongest known non-covalent interactions. Due to its small molecular size, biotin can be covalently conjugated to diverse biomolecules, including proteins, peptides, and nucleic acids, without significantly disrupting their native conformation or biological activity. Biotinylated molecules can then be detected or captured with high specificity in various assay platforms, including enzyme-linked immunosorbent assays (ELISAs), dot blots, and western blots, using streptavidin- or avidin-conjugated probes. For MACS-based yeast display, the antigenic target must initially be biotinylated to enable efficient binding to streptavidin-coated magnetic beads. This biotin–streptavidin interaction facilitates the selective capture and enrichment of yeast cells displaying target-specific binding proteins, thereby enhancing the efficiency of screening and selection in directed evolution or antibody discovery workflows.*

1. Equilibrate the vial of EZ-Link^TM^ Sulfo-NHS-Biotin to room temperature before opening.

2. Dissolve 1–10 mg of protein in 0.5–2 mL of PBS according to calculations in the following equation.

a. Calculate the amount of EZ-Link^TM^ Sulfo-NHS-Biotin (mmol) to add to the reaction for a 20-fold molar excess:

$$mL\;protein\times \frac{mg\;protein}{mL\;protein}\times \frac{mmol\;protein}{mg\;protein}\times \frac{20}{mmol\;protein}=mmol\;biotin$$

Where 20 is the recommended molar fold excess of biotin reagent per protein sample.

b. Calculate the volume of 10 mM EZ-Link^TM^ Sulfo-NHS-Biotin (μL) to add to the reaction:

$$mmol\;biotin\times \frac{443}{mmol\;biotin}\times \frac{500}{2.2}=\mu L\;biotin\;solution$$

Where 443 is the molecular weight of EZ-Link^TM^ Sulfo-NHS-Biotin, and 500 is the volume (µL) of water in which 2.2 mg of Sulfo-NHS-Biotin is dissolved to make 10 mM.

3. Prepare a 10 mM EZ-Link^TM^ Sulfo-NHS-Biotin solution by dissolving 2.2 mg in 500 µL of ultrapure water (sterile, type I) immediately before use.

4. Add the appropriate volume of EZ-Link^TM^ Sulfo-NHS-Biotin solution (as determined in the previous calculation) to the protein solution and mix gently.

5. Incubate the reaction on ice for 2 h or at room temperature for 30–60 min.

*Note: Biotinylation efficiency should be investigated using western blotting with HRP-conjugated streptavidin. After electrophoretic separation and transfer of the biotinylated protein to a membrane, the membrane can be probed with HRP-conjugated streptavidin, which binds specifically to biotin moieties. Detection of the signal via chemiluminescence or colorimetric substrates enables confirmation of successful biotin conjugation, as well as estimation of biotinylation efficiency and protein integrity. This validation step is critical before proceeding to downstream applications such as MACS-based selection.*

**G. Determination of baseline growth in FLI-TRAP screening**

1. Before performing FLI-TRAP selection, the plasmid containing the Tat-signal peptide and Bla-conjugated protein must be constructed. Briefly, the gene of a Ref8 nonspecific Nb gene fragment [7] from the antigen-focused library must be cloned into the pDD18-ssTorA-GCN-4-FLAG plasmid at position GCN-4, which is located between the *Xba*I and *Sal*I restriction sites, to create pDD18-ssTorA-Ref8Nb-FLAG. Simultaneously, clone a PCSK9-catalytic domain (CAT) gene fragment into position Y of the pDD322-TatABC-p17wt-Bla plasmid [5] between the *Avr*II and *Not*I restriction sites to create pDD322-TatABC-CAT-Bla.

2. Transform the constructed plasmids into an *E. coli* strain with a functioning Tat pathway, such as MC4100 or NEB10β. To enhance Tat transport efficiency, the pDD322-TatABC plasmid expresses the TatA, TatB, and TatC proteins from their native promoter. After transformation, plate the transformed cell on LB agar containing 34 μg/mL of chloramphenicol and 50 μg/mL of kanamycin, and incubate the plates at 37 °C overnight. Then, extract and transform the pDD322-TatABC-CAT-Bla plasmid into the cell containing the pDD18-ssTorA-Ref8-FLAG plasmid. Investigate the expression of the ssTorA-Ref8-FLAG and CAT-Bla fusion proteins in further experiments.

3. From the plates incubated overnight, select a single colony and inoculate it into 2 mL of LB medium supplemented with 34 μg/mL of chloramphenicol and 50 μg/mL of kanamycin, and then incubate this culture overnight at 37 °C with shaking at 200 rpm to promote growth.

5. Prepare a series of 100 mm LB agar plates containing increasing concentrations of carbenicillin (from approximately 12 μg/mL to 1 mg/mL) and increasing concentrations of L-arabinose (0.1%, 0.5%, and 1% w/v). Include control plates that contain only increasing concentrations of L-arabinose to monitor nonselective growth. For example, prepare plates containing 0.1% L-arabinose supplemented with 0, 12, 25, 50, or 100 μg/mL of carbenicillin, as well as plates containing 1% L-arabinose and the same carbenicillin concentrations. To prepare these plates, microwave the LB agar medium, cool it to approximately 50 °C in a water bath, and then add the appropriate antibiotics and inducers before pouring 12.5 mL into sterile 100 mm Petri dishes.

6. Measure the OD_600_ of the overnight culture the next morning. Pellet aliquots of the culture by centrifugation, then resuspend and normalize each sample in fresh LB medium to an OD_600_ of 2.5.

7. Using a sterile 96-well plate, prepare an appropriate serial dilution series (we recommend preparing a series of tenfold dilutions from 10^-1^ to 10^-6^) of the normalized cultures. Add 180 μL of fresh LB medium to each well, then add 20 μL of the normalized culture to the first row, creating a tenfold dilution.

8. Spot 5 μL of each dilution onto the prepared LB agar plates, testing all combinations of carbenicillin and L-arabinose concentrations. Use fresh pipette tips for each dilution series to avoid cross-contamination.

9. Allow the spots to dry completely, ensuring no visible droplets remain on the agar surface. Incubate the plates inverted in an incubator and monitor cell growth at intervals of 24–48 h at appropriate temperatures (we recommend 25, 30, and 37 °C based on the temperature that elicits the highest expression of both Ref8Nb and PCSK9-catalytic domain proteins). Image the plates at various time points for later comparison with the library-selected clones. The dilution factor and antibiotic concentration at which growth occurs are associated with the amount of Bla transported by the Tat pathway, which depends on the solubility and affinity of the interacting proteins. The conditions producing the highest background growth caused by Bla leakage from dead cells should be recorded to avoid false-positive clones during FLI-TRAP screening.

*Note: Assess antibiotic resistance under the tested conditions based on the extent of growth on each plate. Additionally, various other parameters of selection conditions need to be optimized, including incubation time, temperature, and inducer concentration. Because each PPI may behave differently, it is advisable to select a range of conditions, including antibiotic concentrations both above and below the ideal level, to cover various interaction strengths.*

**H. Identification of intracellular PPIs through FLI-TRAP library screening**

1. Insert the PCSK9-focused Nb library sequences into position GCN-4 of the pDD18-ssTorA-GCN-4-FLAG plasmid to create pDD18-Nbs-FLAG. Electroporate the plasmid library (≥50 ng/mL) into a high-competency *E. coli* strain, such as NEB10β. To prepare electrocompetent *E. coli* NEB10β cells, prepare five 50 μL aliquots of cells into sterile microcentrifuge tubes and store at -80 °C. Desalt 40 μL aliquots of the plasmid DNA library by drop dialysis on a 0.025 μm nitrocellulose membrane for 20 min. While desalting, thaw the competent cells on ice. For each transformation, combine 30 μL of the plasmid DNA library (10% of the total reaction volume) with five 50 μL aliquots of cells in a 1-mm gap electroporation cuvette. Perform electroporation, then recover the transformed cells by washing three times with 1 mL of prewarmed SOB medium. Transfer the recovered cells into a 250 mL flask. Repeat the transformation process until the entire plasmid library has been introduced. Incubate the cultures at 37 °C with shaking for 1 h.

2. To estimate the number of transformed cells, dilute 20 μL of the culture from the flask into 180 μL of SOB medium in a 1.7 mL microcentrifuge tube. Perform five additional tenfold serial dilutions. Plate 100 μL from each dilution onto LB agar plates containing 34 μg/mL of chloramphenicol. Count the colonies on appropriately diluted plates and scale up to calculate the total number of transformants in the flask, providing an estimate of the library size.

*Note: The library size of pDD18-ssTorA-Nbs-FLAG should be larger than 2 × 10^5^ cells/reaction to increase the possibility of screening. The expression of the Nbs and CAT-Bla proteins must be investigated by western blotting (anti-Flag-HRP for Nbs and anti-Betalactamase antibody for CAT-Bla protein). Both the Nbs and CAT-Bla proteins must be present in the soluble fraction to facilitate the FLI-TRAP selection.*

3. The pDD322-TatABC-CAT-Bla plasmid is subsequently introduced into the *E. coli* library already containing the pDD18-ssTorA-Nbs-FLAG plasmid via electroporation. Briefly, competent cells from the library are thawed on ice, mixed with the pDD322-TatABC-CAT-Bla plasmid, and transferred into an electroporation cuvette. After applying the electric pulse to facilitate uptake of the plasmid DNA, the transformed cells are immediately recovered by adding prewarmed SOB medium and incubating at 37 °C with shaking to allow expression of the resistance marker. This co-transformation enables the simultaneous presence of both plasmids in the host cells, preparing the library for downstream FLI-TRAP screening.

**Critical:** Count the colonies on the serial dilution plates to estimate the total number of transformed clones. We recommend that the number of transformed cells exceeds the number of unique members in the constructed library, ensuring multiple-fold coverage. If the total number of transformed clones is lower than the library size, the transformation should be repeated to achieve sufficient representation of all library members.

4. Prepare LB agar plates supplemented with the appropriate concentrations of carbenicillin and L-arabinose for selection (plate preparation is described in step G5).

5. Measure the OD_600_ of the overnight *E. coli* culture co-harboring the pDD18-ssTorA-Nbs-FLAG and pDD322-TatABC-CAT-Bla plasmids, which will be used for subsequent selection experiments. Transfer aliquots of the culture into centrifuge tubes and pellet the cells by centrifugation. Carefully remove the supernatant and resuspend the cell pellets in fresh LB medium. Adjust each sample to a standardized OD_600_ of 2.5 to ensure uniform cell density across all samples, providing consistent starting conditions for downstream selection and screening procedures.

6. Plate the appropriate dilution of *E. coli* culture co-harboring the pDD18-ssTorA-Nbs-FLAG and pDD322-TatABC-CAT-Bla plasmids on LB agar plates containing appropriate concentrations of carbenicillin and L-arabinose for selection. Incubate the plates at the temperature identified above for 24–48 h (refer to step G9).

7. After growth selection is complete, monitor colony growth for 16–48 h, noting any clones that grow significantly faster than expected. Based on our experience, clones appearing within the first 16 h could represent false positives. Randomly select multiple individual colonies corresponding to potential positive clones and inoculate them into fresh LB medium, followed by incubation at 25 °C overnight for verification and characterization.

**I. Characterization and verification of isolated candidates**

**Figure 3.**
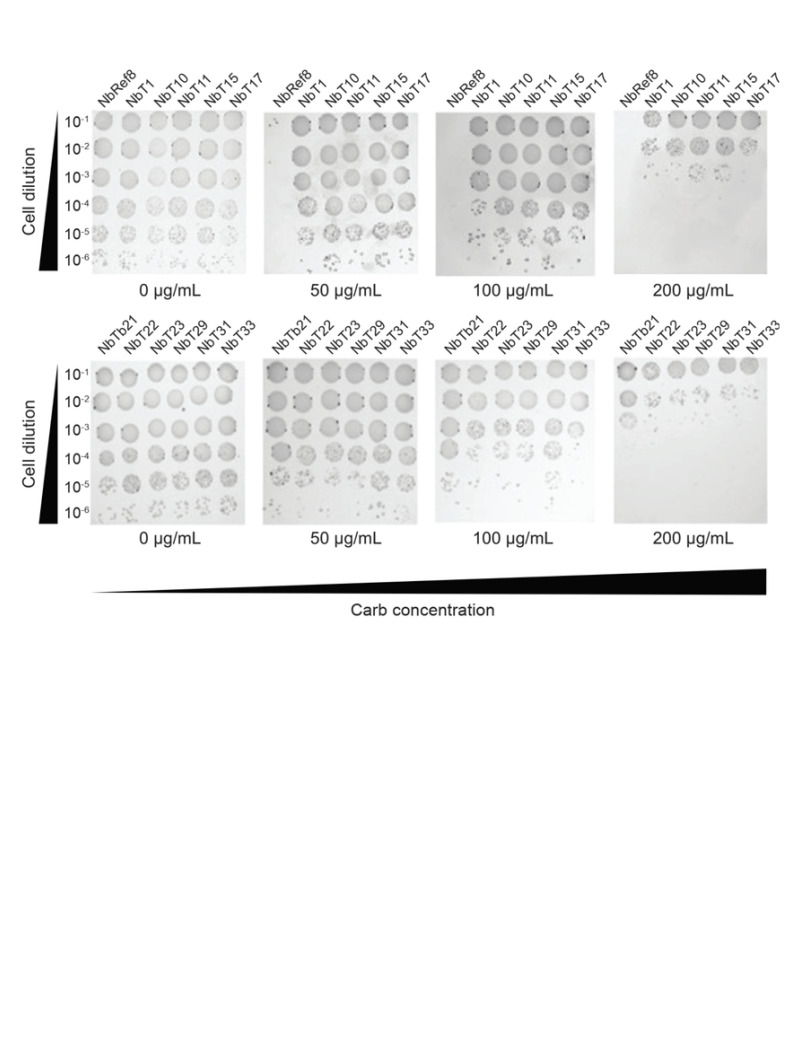
Selection of PCSK9 CAT domain-specific nanobodies (Nbs) performed by co-transforming *E. coli* NEB10β cells with pDD18-ssTorA-Nbs-FLAG and pDD322-CAT-Bla on LB agar containing 0–300 µg/mL carbenicillin (Carb). Thirty random resistant colonies were isolated, and sequencing identified 11 unique full-length nanobody sequences. Spot plating analysis showed that all 11 clones conferred strong resistance at 50–100 µg/mL carbenicillin, with reduced but detectable resistance at 200 µg/mL. In contrast, the clone containing pDD15-Ref8-FLAG nanobody did not support cell growth at 50 µg/mL [7].

1. Serially dilute the isolated clones and spot-plate them under the same selection conditions used for their initial isolation (repeat steps G5–9). Include a parental plasmid control for each tested condition.

2. Select clones exhibiting higher carbenicillin resistance for plasmid extraction (Figure 3), followed by sequencing to identify mutations in positive clones. Sequencing the full length of the mutated gene will confirm the presence of the desired insert and verify the absence of frameshifts or other unintended alterations.

**Critical:** The phenotype of positive clones must be carefully verified to prevent false positives arising from *E. coli* clones that acquire spontaneous carbenicillin tolerance. Plasmids from the initially identified positive clones should be extracted and reintroduced into new *E. coli* cells [e.g., MC4100, NEB10 β, or BL21(DE3)]. These transformed cells should then be spot-plated under the same selection conditions. True positives are confirmed when the reintroduced plasmid confers the same antibiotic resistance observed in the initially isolated library clones, ensuring the phenotype is plasmid-dependent rather than host-derived.

3. Subclone the selected clones into the pET28a expression vector to achieve higher levels of protein expression for in vitro analysis. Investigate the expressed Nbs in a series of biochemical and biophysical assays to evaluate their specificity and binding affinity. Techniques such as western blotting can be employed to confirm the presence and correct size of the expressed proteins, while ELISAs can be utilized to quantify target-specific binding activity. Additionally, surface plasmon resonance analysis can be used to provide real-time measurements of binding kinetics. Collectively, these experiments enable the comprehensive characterization of the Nb clones, confirming their functional activity and suitability for downstream applications. As shown in Figure 4 of Thaiprayoon et al. [7], further evaluation of the binding potential of the selected nanobodies against their protein targets remains necessary, since both in vivo and in vitro interaction properties require more definitive validation [7].

## Validation of protocol

This protocol provides a general strategy for selecting and validating PPIs from combinatorial libraries, such as Nb or other small binding protein libraries, against arbitrary target proteins. The workflow combines MACS-based yeast display library enrichment with genetic selection using the FLI-TRAP system. MACS enables rapid pre-selection of library members that bind a target of interest, while FLI-TRAP allows further enrichment of clones with high-affinity and correctly folded interactions in vivo by leveraging the Tat-dependent export quality control.

**General applicability and reproducibility**

1. The protocol can be applied to a wide range of soluble protein pairs, where the first protein (library member) is displayed or expressed in a host cell (e.g., yeast or bacteria) and the second protein (target) is either biotinylated for MACS enrichment or fused to a reporter for genetic selection.

2. MACS enrichment reliably reduces library complexity while retaining diversity of potential binders, and FLI-TRAP selection efficiently removes misfolded or nonspecific binders by correlating host cell survival with protein–protein binding strength.

**In vitro validation using PCSK9 and selected nanobodies as an example**

To demonstrate the effectiveness of this general protocol, it was applied to select Nbs targeting the catalytic (CAT) domain of human PCSK9. The following in vitro validation assays confirmed the reliability of the workflow:

1. Expression and structural integrity (western blot and SDS-PAGE): Selected Nbs (15–17 kDa) were expressed in *E. coli* and purified. Western blot and SDS-PAGE confirmed expected molecular weights and integrity, demonstrating that enriched clones maintained proper expression and stability.

2. Binding specificity and dose-dependent interaction (ELISA): Purified Nbs were tested for binding to full-length wild-type PCSK9 and a gain-of-function mutant (PCSK9^D374Y^). ELISA revealed specific binding of selected Nbs, with signals significantly above that of nonspecific controls, such as bovine serum albumin. Dose-response curves confirmed concentration-dependent binding, illustrating that clones enriched via MACS and FLI-TRAP combination retained high-affinity target recognition.

3. Functional validation (inhibition of protein–protein interaction): Selected Nbs inhibited the interaction between PCSK9 and LDL receptor (LDLR) in a dose-dependent manner. NbT22, for example, exhibited the most potent inhibition, demonstrating that enriched binders are biologically active and capable of modulating target interactions.

4. Statistical analysis and controls: All assays were performed in triplicate, and mean ± standard deviation values were reported. Dose-response ELISA inhibition assays included calculation of 95% confidence intervals. Negative controls, such as non-binding Nbs or lysates from host cells lacking the library construct, confirmed the specificity of selected binders. This protocol provides a generalizable and reproducible workflow for isolating and validating high-affinity protein–protein interaction pairs from combinatorial libraries. The MACS-based enrichment step allows rapid initial selection, while FLI-TRAP provides in vivo folding quality control and affinity-based selection. In vitro validation assays, including western blot and ELISA, confirm that the selected clones are structurally intact, specific, and functionally active. The selection of PCSK9-binding nanobodies in previous studies demonstrates the effectiveness of this protocol and provides a concrete example of its application.

**This protocol (or parts of it) has been used and validated in the following research article(s):**

Apisitt Thaiprayoon et al. [7]. Isolation of antigen-specific nanobodies from synthetic libraries using a protein selection strategy that combines MACS-based screening of YSD and FLI-TRAP. *Scientific Reports* (Figures 2–7).

## General notes and troubleshooting

**General notes**

1. Applicability to other protein systems: Although this protocol was validated using Nbs and PCSK9 interactions, the general workflow can be adapted for other protein–protein partners. Users should optimize expression systems, linker designs, and tagging strategies according to the specific proteins being studied.

2. Library complexity and coverage: The efficiency of selection depends on the diversity and representation of the library. For large libraries (>10^8^ variants), multiple rounds of MACS or genetic selection may improve recovery of rare high-affinity binders.

3. In vivo folding quality control: FLI-TRAP relies on the Tat system to export properly folded complexes. Proteins prone to misfolding may be underrepresented. Consider using molecular chaperones or alternative expression hosts if folding issues are suspected.

4. Reproducibility considerations: Variability can arise from differences in cell density, antigen labeling efficiency, or magnetic bead batch quality. Include negative and positive controls in each experiment to monitor consistency.

5. Downstream validation: Clones enriched using this protocol should be confirmed using in vitro assays such as western blot, ELISA, or surface plasmon resonance (SPR) to verify structural integrity, binding specificity, and functional activity (see Validation section).

**Troubleshooting**

**Problem 1:** Low recovery of library members after MACS enrichment.

**Possible causes:**

• Inefficient biotinylation of the target protein.

• Low concentration of antigen during incubation.

• Poor surface expression of library members.

• Magnetic bead aggregation or insufficient mixing.

**Solutions:**

• Optimize antigen biotinylation conditions (verify by western blot with streptavidin-HRP).

• Increase target protein concentration or incubation time.

• Confirm induction of library proteins (yeast Nb expression) before MACS.

• Vortex beads thoroughly and ensure homogeneous mixing.

**Problem 2:** High background growth in FLI-TRAP selection.

**Possible causes:**

• Leaky expression of the Bla reporter without proper Nbs and target interaction.

• Nonspecific interactions between library members and cellular proteins.

• Suboptimal antibiotic concentration that fails to discriminate between strong and weak interactions.

**Solutions:**

• Titrate antibiotic concentration to select for only strong interactions.

• Include negative controls (library cells with irrelevant antigen) to measure baseline growth.

• Use multiple rounds of selection and antibiotic titration if necessary to enrich true binders.

**Problem 3:** Selected clones express insoluble or misfolded proteins in vitro.

**Possible causes:**

• Protein may misfold after heterologous expression despite proper folding in FLI-TRAP.

• Expression conditions (temperature, host strain) are not optimal for soluble protein production.

**Solutions:**

• Test alternative expression systems (e.g., *E. coli* strains with chaperones, yeast, or mammalian cells).

• Include solubility tags (MBP, SUMO, GST) or use lower induction temperatures.

• Verify protein integrity with SDS-PAGE, western blot, ELISA, or SPR.

## References

[1] Lu R. M., Hwang Y. C., Liu I. J., Lee C. C., Tsai H. Z., Li H. J. and Wu H. C. (2020). Development of therapeutic antibodies for the treatment of diseases. J Biomed Sci. 27(1): e1186/s12929–019–0592–z. 10.1186/s12929-019-0592-z PMC6939334 31894001

[2] Carter P. J. and Rajpal A. (2022). Designing antibodies as therapeutics. Cell. 185(15): 2789 2805 2805. 10.1016/j.cell .2022.05.029 35868279

[3] J. Yong Joon Kim, Sang Z., Xiang Y., Shen Z. and Shi Y. (2023). Nanobodies: Robust miniprotein binders in biomedicine. Adv Drug Delivery Rev. 195: 114726 10.1016/j.addr .2023.114726 PMC11725230 36754285

[4] Jin B. k., Odongo S., Radwanska M. and Magez S. (2023). NANOBODIES®: A Review of Diagnostic and Therapeutic Applications. Int J Mol Sci. 24(6): 5994 10.3390/ijms24065994 36983063 PMC10057852

[5] Waraho D. and DeLisa M. P. (2009). Versatile selection technology for intracellular protein–protein interactions mediated by a unique bacterial hitchhiker transport mechanism. Proc Natl Acad Sci USA. 106(10): 3692 3697 3697. 10.1073/pnas.0704048106 19234130 PMC2656142

[6] Waraho-Zhmayev D., Meksiriporn B., Portnoff A. D. and DeLisa M. P. (2014). Optimizing recombinant antibodies for intracellular function using hitchhiker-mediated survival selection. Protein Eng Des Sel. 27(10): 351 358 358. 10.1093/protein/gzu038 25225416 PMC4191445

[7] Thaiprayoon A., Chantarasorn Y., Oonanant W., Kasorn A., Longsompurana P., Tapaneeyakorn S., Riangrungroj P., Loison F., Kruse A. C., DeLisa M. P., .(2025). Isolation of PCSK9-specific nanobodies from synthetic libraries using a combined protein selection strategy. Sci Rep. 15(1): 3594 10.1038/s41598-025-88032-1 39875480 PMC11775271

[8] Taw M. N., Boock J. T., Sotomayor B., Kim D., Rocco M. A., Waraho-Zhmayev D. and DeLisa M. P. (2022). Twin-arginine translocase component TatB performs folding quality control via a chaperone-like activity. Sci Rep. 12(1): 14862 10.1038/s41598-022-18958-3 36050356 PMC9436932

[9] Intasurat K., Submunkongtawee N., Longsompurana P., Thaiprayoon A., Kasemsukwimol W., Sirimanakul S., Boonsilp S., Seetaha S., Choowongkomon K., Waraho-Zhmayev D., .(2024). Redirecting a Broad-Spectrum Nanobody Against the Receptor-Binding Domain of SARS-CoV-2 to Target Omicron Variants. Appl Sci. 14(22): 10548 10.3390/app142210548

[10] McMahon C., Baier A. S., Pascolutti R., Wegrecki M., Zheng S., Ong J. X., Erlandson S. C., Hilger D., Rasmussen S. G. F., Ring A. M., .(2018). Yeast surface display platform for rapid discovery of conformationally selective nanobodies. Nat Struct Mol Biol. 25(3): 289 296 296. 10.1038/s41594-018-0028-6 29434346 PMC5839991

